# Effects of posed smiling on memory for happy and sad facial expressions

**DOI:** 10.1038/s41598-021-89828-7

**Published:** 2021-05-18

**Authors:** Maria Kuehne, Tino Zaehle, Janek S. Lobmaier

**Affiliations:** 1 Department of Social Neuroscience and Social Psychology, Institute of Psychology, University of Bern, Bern, Switzerland; 2 Department of Neurology, Otto-Von-Guericke-University Magdeburg, Leipziger Straße 44, 39120 Magdeburg, Germany; 3 Center for Behavioral Brain Sciences, Magdeburg, Germany

**Keywords:** Human behaviour, Cognitive neuroscience, Emotion

## Abstract

The perception and storage of facial emotional expressions constitutes an important human skill that is essential for our daily social interactions. While previous research revealed that facial feedback can influence the perception of facial emotional expressions, it is unclear whether facial feedback also plays a role in memory processes of facial emotional expressions. In the present study we investigated the impact of facial feedback on the performance in emotional visual working memory (WM). For this purpose, 37 participants underwent a classical facial feedback manipulation (FFM) (holding a pen with the teeth—inducing a smiling expression vs. holding a pen with the non-dominant hand—as a control condition) while they performed a WM task on varying intensities of happy or sad facial expressions. Results show that the smiling manipulation improved memory performance selectively for happy faces, especially for highly ambiguous facial expressions. Furthermore, we found that in addition to an overall negative bias specifically for happy faces (i.e. happy faces are remembered as more negative than they initially were), FFM induced a positivity bias when memorizing emotional facial information (i.e. faces were remembered as being more positive than they actually were). Finally, our data demonstrate that men were affected more by FFM: during induced smiling men showed a larger positive bias than women did. These data demonstrate that facial feedback not only influences our perception but also systematically alters our memory of facial emotional expressions.

## Introduction

In human social interactions, facial expressions play an important role. Facial expressions transmit internal states such as motivations and feelings, which makes them an important source of non-verbal information. Various studies indicate that facial expressions are mimicked by eliciting facial muscular activity congruent with the presented facial expressions^1,2^. Generally, this mimicry process appears to be automatic and can occur without attention^3^. Theories of embodied simulation assume that the mimicked facial expression and the resulting feedback from the facial muscles trigger a corresponding state in the observer’s motor, somatosensory, affective and reward system, helping to decode and understand the meaning of the perceived expression^4^. These mimicry processes seem to be implemented differently in men and women^5^. Early research has shown that women are emotionally more expressive^6^ and show more mimicry^7^ than men. More recently, Niedenthal and colleagues showed that the duration of pacifier use in early childhood negatively impacts facial mimicry in boys but not in girls and additionally, that this pacifier use is correlated with emotional intelligence and perspective taking skills in men’s later life^8^. The authors interpret these findings such that the usage of a pacifier leads to a reduction of facial mimicry processes in the user as well as to reduced facial mimicry reactions towards the user. While girls are thought to compensate for these negative consequences, boys are thought to be at the mercy of these consequences^9^.

While some studies investigated facial mimicry using electromyographic measures^2,10–12^, others have experimentally manipulated the facial feedback processes to investigate its impact on the processing of emotional stimuli^13–15^. The classical facial feedback manipulation method was first introduced by Strack et al. in 1988^16^. Here, participants are asked to hold a pen in their mouth, in different ways. The underlying principle behind this approach is that different pen holding conditions differentially activate facial muscles essential for smiling. In particular, when participants hold the pen with their teeth they activate the Musculus zygomaticus major and the Musculus risorius—both these muscles are activated while smiling. In contrast, when participants hold the pen with their lips they activate the Musculus orbicularis oris, the contraction of which is incompatible with smiling. Notwithstanding an ongoing intense debate about the replicability of the seminal study by Strack and colleagues^17–20^, there is ample evidence that such facial feedback manipulations influence the conscious processing of emotional facial expressions^13,21,22^ and emotional body expressions^23^, as well as the automatic processing of unattended facial emotional expressions^24^. Recently, we investigated the impact of facial feedback on the automatic processing by electrophysiological measurements of the expression-related mismatch negativity (eMMN). Facial feedback manipulation was implemented by different pen holding conditions equivalent to the study by Strack and colleagues^16^. While the results demonstrated that in particular the smiling condition differentially influenced the automatic processing of happy and sad facial expressions, the affected underlying cognitive process remains elusive. We assumed that the facial feedback manipulation influenced the encoding and retrieval of happy and sad facial expressions. Specifically, we interpreted these findings such that the smiling manipulation condition might have facilitated the encoding of happy while impeding the encoding of the sad faces. Therefore, the emotional valence of the happy face might have been stored more effectively than sad faces^24^.

To date, only very few studies have looked at the influence of facial mimicry and the resulting facial feedback on the storage and retrieval of facial emotional expressions. One recent study by Pawling et al.^25^ demonstrated that the visual re-exposure to a facial expression reactivated the corresponding mimicry in a similar way as did the initial exposure. Interestingly, this emotional mimicry re-activation also occurred when the same face identity was displayed with a neutral expression during the re-exposure. These results are in accordance with the reactivation account of memory, indicating that the same brain regions are activated during retrieval and encoding (for review see Danker and Anderson^26^).

The present study examined the role of facial feedback in memory processes of emotional facial expressions in a facial feedback manipulation study using an emotional working memory task. Facial feedback manipulation was administered following Strack et al.^16^. However, as previous studies have shown no or only minor effects of the holding-the-pen-between-the-lips-manipulation^13,24^ we restricted our manipulation to the smile-inducing condition, in which participants hold a pen with their teeth. We compared this manipulation to a neutral control condition (holding the pen with the non-dominant hand). Memory performance was investigated using a modified emotional working memory (WM) paradigm with facial expressions, allowing us to separate overall WM accuracy from emotional biases^27^. The participants’ task was to encode, maintain and subsequently retrieve the valence as well as the intensity of happy and sad faces. In accordance with Mok and colleagues^27^ we predicted that the intensity of the expressions will affect memory, with better performance for less ambiguous emotional expressions. Further, we expected the teeth-manipulation to increase facial feedback of smiling, which in turn affects memory performance. Finally, following findings suggesting gender effects on recognition of emotional faces^28,29^ as well as on facial mimicry^5–9^, we assumed that memory performance might differ between women and men.

## Methods

### Participants

We investigated 37 healthy participants (19 women, 18 men, mean age 25 years ± 3.42). Sample size was determined based on previous studies^24,27,30^.

Participants had normal or corrected to normal vision and had no history of neurological or neuropsychiatric diseases. After arriving at the laboratory, all participants provided written informed consent and filled in the short version of the *Allgemeine Depressionsskala* (ADS-K, self-report questionnaire measuring impairments due to depressive symptoms during the previous weeks^31^). Additionally, participants were asked to complete the *Implicit Positive and Negative Affect Test* (IPANAT, measuring implicit positive and negative affect as well as state variance^32^). They filled in the IPANAT three times, before, during and after the experiment. All sample characteristics are presented in Table 1. The study and its experimental procedures were conducted in accordance with the Declaration of Helsinki (1991; p. 1194) and were approved by the local Ethical Committee of the University of Magdeburg.

**Table 1:** Sample characteristics.

Measure (n = 37 )	M(SD)	Range
Age	25 (3.42)	18–34
ADS-K	7.35 (3.92)	1–15
**IPANAT**		
1	2.15_PA_ (0.39)	1–3
	1.82_NA_ (0.51)	1–3
2	2.24_PA_ (0.41)	2–3
	1.68_NA_ (0.42)	1–3
3	2.15_PA_ (0.48)	1–3
	1.77_NA_ (0.38)	1–3

Age in years, ADS-K, IPANAT before (1), during (2) and after (3) the emotional WM task, separately for positive affect (PA) and negative affect (NA). Additional analysis with ADS-K as well as with IPANAT is documented in supplementary material.

### Stimuli and procedure

**Figure 1:**
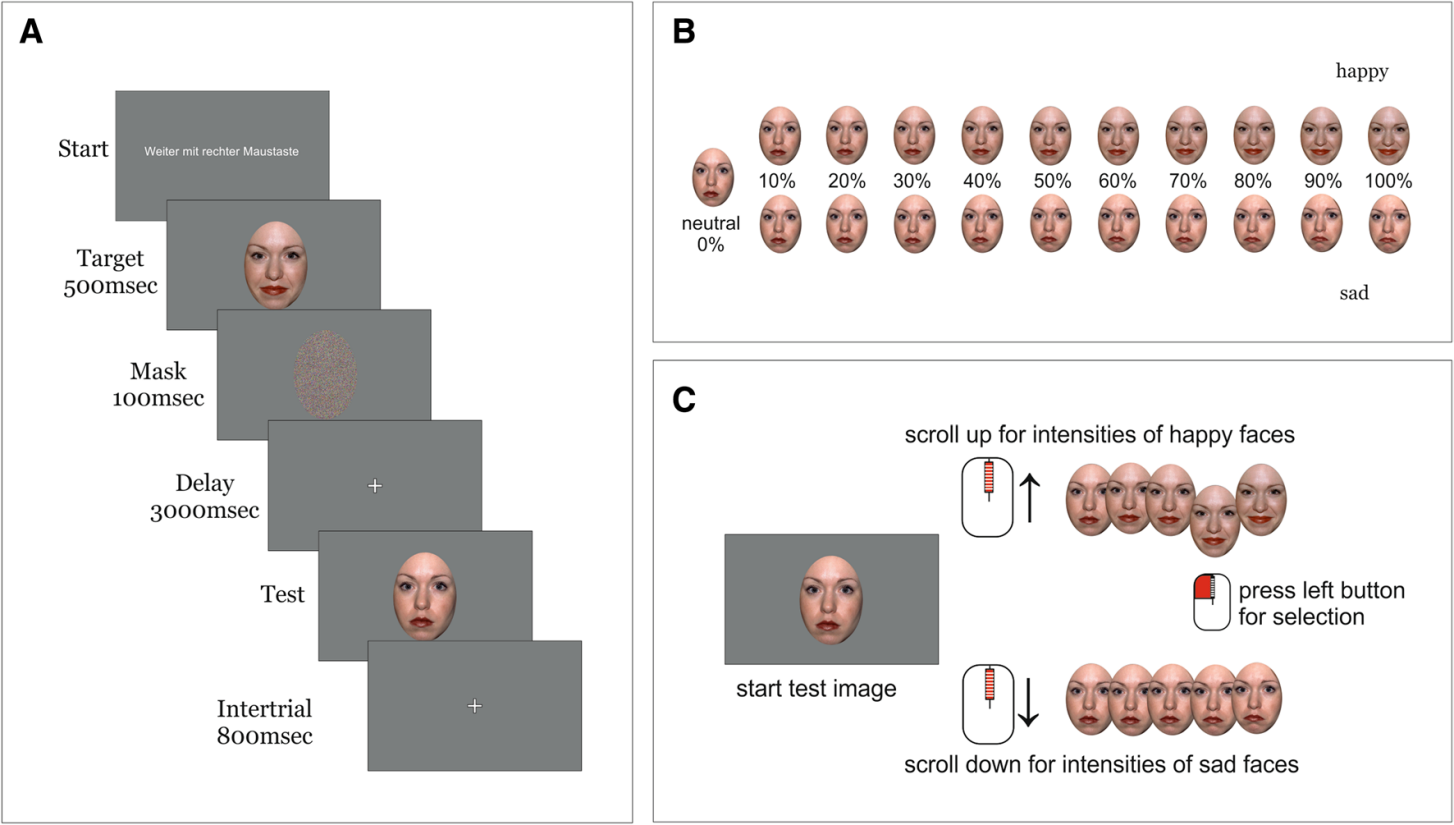
Procedure of the facial emotion WM task. On each trial, participants were asked to encode a target face with an emotional expression (sad or happy) of a certain intensity. After a delay, participants used a mouse to adjust the expression to match the emotion and intensity of the face in memory. (**A**) Trial example. Each trial began with a starting image, presented until participants press the right mouse button. The target image was displayed for 500 ms followed by a mask image of 100 ms. After a delay of 3000 ms the test image was shown and participants had to respond. After the response or after 11 s a fixation cross appeared for 800 ms before the next trial started. (**B**) Target Image. The target image was either a happy or a sad emotional face at one of 11 intensity steps (neutral, 10, 20, 30, 40, 50, 60, 70, 80, 90, 100% sad or happy). (**C**) Test Image. The test image always started with a neutral face. By using the mouse wheel, participant could adjust the remembered emotion and the intensity. Scrolling the mouse wheel changed the intensity of the emotional face continuously in steps of 1%. By pressing the left mouse button, the participant made their final selection.

At the beginning, participants read the instruction of the task and filled in the questionnaires. The experiment proper consisted of an emotional WM task (adapted from Mok et al.^27^). Six female and seven male characters, each with three different emotional expressions (neutral, happy and sad), were taken from the NimStim Set of Facial Expressions^33^ (Stimulus IDs: 01F, 02F, 03F, 05F, 07F, 09F, 20M, 21M, 23M, 29M, 32M, 34M) and from the Karolinska face data-base^34^ (Stimulus ID: AM14). The stimuli were equally distributed to the different pen holding conditions (3male/female for hand and 3 male/female for teeth; 1 male, 29M, for practice). All stimuli were edited with GIMP software (Version 2.10.6). To avoid low-level visual influence the hair region of each character was cut out by putting an eliptic shape around the head with grey background. From this elliptic shape, a scrambled mask was created separately for each character by changing pixels into random colors thereby producing white noise (see Fig. 1a). To familiarize the participants with the emotional WM task, they performed a practice trial before starting the main task. During the emotional WM task, participants had to encode, retrieve and maintain the emotion itself and the specific intensity of an emotional face while holding a pen either between their teeth or with the non-dominant hand. The two pen holding conditions alternated over 12 different blocks. Each block consisted of 21 trials (overall 126 trials for each pen holding condition). Each trial began with a starting screen lasting until participants pressed the right mouse button. Thereafter the target image appeared for 500 ms, followed by the mask for 100 ms. After a delay of 3000 ms, the test image was shown and participants gave their response (see below). After an interval of 800 ms, the next trial started (see Fig. 1a). The target image displayed a face with a specific intensity of either happy or sad emotion. For this purpose, morph sequences were created from neutral to happy and from neutral to sad expressions in 1-degree-steps from 0 to 100% using java psychomorph^35^ (version 6). For target images, intensities in 10% steps were used (0% happy/sad, 10% happy/sad, 20% happy/sad, 30% happy/sad, 40% happy/sad, 50% happy/sad, 60% happy/sad, 70% happy/sad, 80% happy/sad, 90% happy/sad, 100% happy/sad, see Fig. 1b). During the task, each character was presented with each intensity step as target image. The test image was always the neutral face of the character. By scrolling the mouse wheel back and forth, participants could adjust the emotion and the intensity of the emotion to match the memorised target face. All intensity levels from 0–100% were possible for the response selection (see Fig. 1c). The response time window was restricted to 11 s. There were 8 different versions of the task, varying the order of pen holding conditions (starting with hand or teeth), identity allocation to pen holding conditions and mouse wheel settings (scroll up: face becomes happier, scroll down: face becomes sadder or vice versa). The Versions were pseudorandomly assigned to the participants.

### Data analysis

To investigate the influence of facial feedback manipulation (FFM) on emotional memory we assessed the quality of WM representations for emotional facial expressions and the systematic affective biases in perceiving and interpreting these expressions^27^. Accordingly, we separately analyzed performance accuracy (categorical judgment of a happy or sad face) and emotional bias (remembered emotional expression is more positive or more negative than the original) for the two pen holding conditions and the two facial emotions. Trials with neutral target faces were excluded from this analysis.

To characterize the accuracy of WM performance, we assessed the percentage of correct responses. A response was considered correct when participant adjusted a face to the correct emotion type (e.g., reporting a happy face as happy and a sad face as sad). To analyze the effect of ambiguity, the intensity levels were median-split in two equal bins of high and low ambiguity and percent correct responses were computed for each target emotion intensity bin. Mean percent correct responses were entered into a repeated measures (RM) -ANOVA with the within-participant factors *FFM* (hand vs. teeth), *emotion* (happy vs. sad) and *ambiguity* (high vs. low) and the between-participant factor *gender* (male vs. female). Additionally, an ANOVA including all intensity levels was performed.

The emotional bias represents the signed percentage deviation of the test image from the target image, such that negative values imply that participants remembered the emotion as less positive/more negative than the target image originally was and positive values imply that they remembered it as more positive/less negative (see supplementary material for formulas). Consequently, an emotional bias of − 5% would indicate that a target image is remember 5% less positive/more negative than it originally was. After calculating the percentage deviation, an outlier analysis was performed on individual level for each participant separately for the two pen holding conditions (hand, teeth) and the two emotion conditions (happy, sad). Values exceeding ± 2 standard deviations from the mean were excluded from further analysis. This resulted on average in 2.73 (± 1.03 SD) excluded trials for happy and 3.32 (± 1.01 SD) for sad faces during the hand and 2.65 (± 1.12 SD) excluded trials for happy and 3.05 (± 1.29 SD) for sad faces during the teeth condition (see supplementary material for more details). Data of the emotional bias were entered into a RM- ANOVA with the within-participant factors *FFM* (hand vs. teeth), *emotion* (happy vs. sad) and between-participant factor *gender* (male vs. female). If necessary, Greenhouse–Geisser adjustment was used to correct for violations of sphericity. All significant interactions were post-hoc examined by using paired *t* tests and Bonferroni family-wise error correction was applied. The statistical analysis was performed by using IBM SPSS (version 26).

## Results

### Accuracy of WM performance

**Figure 2:**
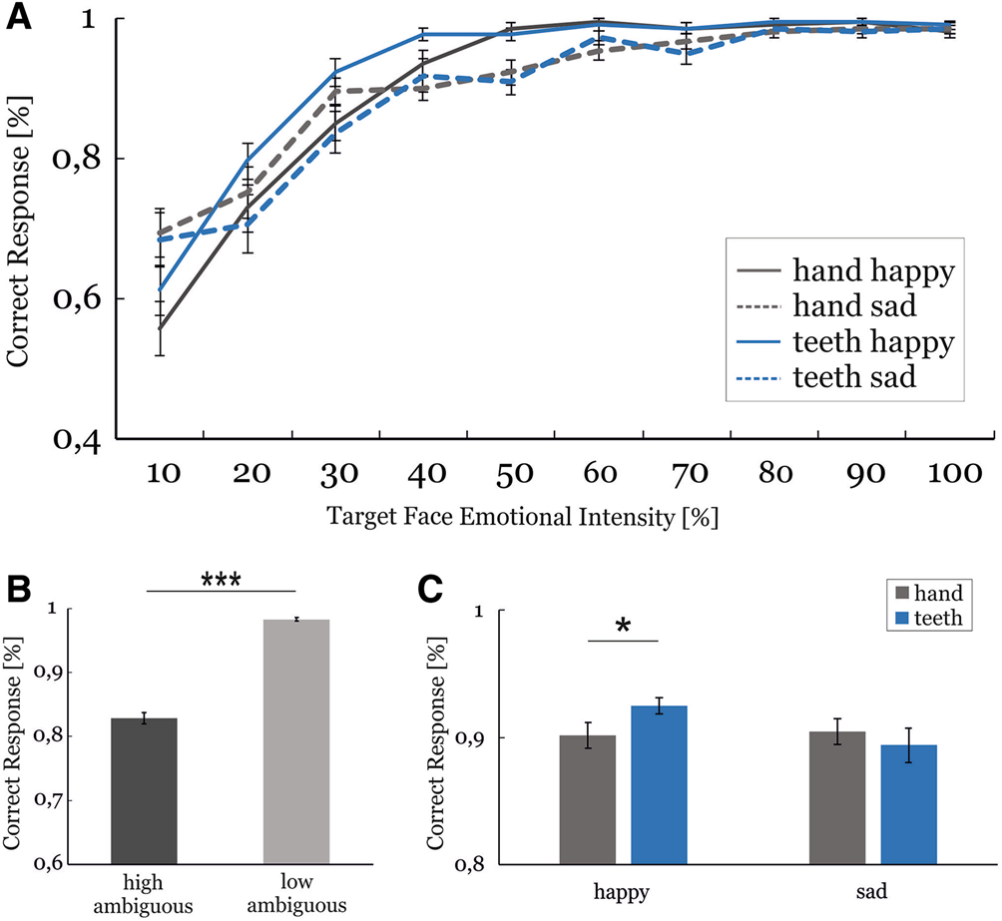
Accuracy of WM Performance. (**A**) Percent correct responses for each emotional intensity of the target face during the hand condition for happy (solid grey) and sad (dashed grey) faces and during the teeth condition for happy (solid blue) and sad (dashed blue) faces. (**B**) Percent correct responses for highly (left) and less (right) ambiguous emotional faces. In comparison to less ambiguous faces, memory accuracy for highly ambiguous faces was significantly reduced. (**C**) Percent correct responses across all intensity levels for happy (left) and sad (right) faces during the hand (grey) and teeth (blue) FFM condition. Whereas FFM did not influence the memory accuracy to sad faces, FFM improved the accuracy for happy faces during the teeth condition. Happy faces were more often correctly remembered in the teeth compared to the hand condition. Error bars represent standard errors (SE). ***p < .001, *p < .05.

Figure 2A illustrates the percentage of correct responses for each emotional intensity separately for both emotions and FFM conditions. The RM-ANOVA revealed a significant main effect of the factor *ambiguity* (F_1,35_ = 487,407, *p* < 0.001, $\eta_{p}^{2}$ = 0.933). As can be seen in Fig. 2B, memory accuracy was reduced for more ambiguous faces (faces with low intensity levels, M = 0.83, SD = 0.05). Specifically, more ambiguous faces were more often incorrectly remembered as expressing the wrong emotion than faces with a more explicit emotion (high intensity levels, M = 0.98, SD = 0.02). The RM-ANOVA further revealed a significant *FFM* × *emotion* interaction (F_1,35_ = 4.293, *p* = 0.046, $\eta_{p}^{2}$ = 0.109). Post-hoc comparisons showed that, compared to the hand condition, the teeth condition significantly increased the accuracy of happy faces only (M_hand_ = 0.90, SD_hand_ = 0.06, M_teeth_ = 0.92, SD_teeth_ = 0.04, t(36) = − 2.537, *p* = 0.016, d = − 0.392). There was no effect of *FFM* on correct responses to sad faces (M_hand_ = 0.90, SD_hand_ = 0.06, M_teeth_ = 0.89, SD_teeth_ = 0.08, t(36) = 0.808, *p* = 0.424, d = 0.141, see Fig. 2C). Finally the ANOVA revealed a significant *FFM* × *emotion* × *ambiguity* interaction (F_1,35_ = 4.429, *p* = 0.043, $\eta_{p}^{2}$ 0.112). A subsequent step-down analysis by means of the factor *ambiguity* revealed a significant *FFM *×* emotion* interaction (F_1,36_ = 4.447, *p* = 0.042, $\eta_{p}^{2}$ = 0.110) for highly ambiguous emotional faces. This interaction is explained by a significant increase of correct responses in the teeth compared to the hand condition for happy (M_hand_ = 0.81, SD_hand_ = 0.11, M_teeth_ = 0.86, SD_teeth_ = 0.08, t(36) = − 2.665, *p* = 0.011, d = − 0.520) but not for sad faces (M_hand_ = 0.83, SD_hand_ = 0.10, M_teeth_ = 0.81, SD_teeth_ = 0.14, t(36) = 0.909, *p* = 0.369, d = 0.164,). In contrast, the RM-ANOVAs for the less ambiguous emotional faces revealed no significant effect of the factor *FFM* or its interactions (all ps > 0.8). The results were comparable when including all 10 levels of *intensity*, showing a significant main effect of the factor *intensity level* (F_3.907,136.737_ = 211.870, *p* < 0.001, $\eta_{p}^{2}$ = 0.858), a significant *FFM* × *emotion* interaction (F_1,35_ = 4.293, *p* = 0.046, $\eta_{p}^{2}$ = 0.109) and a marginally significant *FFM* × *emotion* × *intensity level* interaction (F_3.779, 132.268_ = 2.323, *p* = 0.063, $\eta_{p}^{2}$ = 0.062).

### Emotional bias

**Figure 3:**
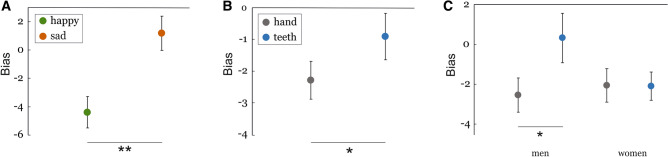
Emotional bias in the working memory task. (**A**) Effect of emotion. Happy faces (green) were remembered as being less happy (i.e., more negative) for sad faces this negative bias was less pronounced. (**B**) Effect of FFM. Independent of the emotion, emotional faces were remembered as being more positive/less negative during teeth (blue) than during the hand (grey) condition. (**C**) Emotional bias for male (left side) and female (right side) participants for the hand (grey) and teeth (blue) FFM condition. Men remembered faces more positive during the teeth compared to the hand condition than did women. Error bars represent standard errors (SE). *p < .05, **p ≤ .01.

Figure 3 illustrates the results for the emotional bias. The RM-ANOVA revealed significant main effects of the factors *FFM* (F_1,35_ = 5.010, *p* = 0.032, $\eta_{p}^{2}$ = 0.125) and *emotion* (F_1,35_ = 7.288, *p* = 0.011, $\eta_{p}^{2}$ = 0.172) as well as a significant interaction between *FFM* and *gender* (F_1,35_ = 5.260, *p* = 0.028, $\eta_{p}^{2}$ = 0.131). The main effect of *emotion* results from a overall more negative bias for happy faces (M = − 4.39, SD = 6.88) compared to sad faces (M = 1.19, SD = 7.47, t(36) = − 2.738, *p* = 0.01, d = 0.778) (see Fig. 3A). Happy faces were remembered as less positive/more negative than their respective target images were. The main effect of *FFM* is shown in Fig. 3B. Independent of the emotion, the teeth condition reduced the negative bias, that is, faces were remembered to be more positive/less negative when participants held the pen with their teeth (M = − 0.91, SD = 4.54) compared to the hand condition (M = − 2.29, SD = 3.72, t(36) = − 2.057, *p* = 0.047, d = − 0.333).

To further examine the *FFM* × *gender* interaction post hoc comparisons between the hand and the teeth conditions were conducted separately for male and female participants. While in men the teeth condition significantly lowered the negative bias compared to the hand condition (M_hand_ = − 2.54, SD_hand_ = 4.27, M_teeth_ = 0.22, SD_teeth_ = 5.62, t(17) = − 2.473, *p* = 0.024, d = − 0.553) there was no influence of the FFM in women (M_hand_ = − 2.37, SD_hand_ = 4.70, M_teeth_ = − 2.16, SD_teeth_ = 3.81, t(18) = − 0.295, *p* = 0.771, d = − 0.049; see Fig. 3C).

## Discussion

Several studies have shown that facial muscle manipulation—and thus facial feedback manipulation—influences conscious as well as automatic processing of emotional faces, both on a behavioral and electrophysiological level. Yet, these studies did not ascertain whether the link between FFM and emotion processing are solely attributable to influences on a perceptual level or whether other cognitive processes are altered as well. To investigate the impact of FFM on memory processes for emotional faces we deployed a facial emotional working memory (WM) paradigm while participants held a pen either between their teeth or with the non-dominant hand. Holding a pen between the teeth leads to activating facial muscles used when smiling (smiling manipulation) and thus to an increased positive facial feedback whereas holding the pen in the non-dominant hand serves as control condition (no FFM). Overall, our data show that the smiling manipulation condition improved the memory performance selectively for happy faces, especially when highly ambiguous facial expression had to be remembered. Furthermore, we found that happy faces were remembered as being less happy (negative bias). For sad faces, we found no bias. Interestingly, the teeth manipulation (smiling feedback) resulted in less negative and more positive memory of emotional facial information. Finally, our data demonstrate that men were more strongly affected by the FFM than women; during smiling manipulation men showed a reduced negative bias.

The data of the present study are in line with the results of previous reports using a comparable WM design^27^. As anticipated, the emotional faces with low intensity/high ambiguity were more often falsely remembered as the wrong emotion than faces with more clear expressions. This can be explained by the fact that facial expressions of low intensities are generally more difficult to recognize. Comparable recognition difficulties of low-intensity emotional expressions have also been reported by Montagne and colleagues in 2007^36^. We found a pronounced influence of the FFM particularly for high ambiguous faces; this is in line with theories of embodied cognition which assume that facial mimicry and its resulting facial feedback contribute to facial emotion recognition, especially when the senders’ expressions is ambiguous^37^.

Furthermore, our data revealed a stronger negative bias for happy faces than for sad faces. A negative memory bias was also reported by Mok and colleagues^27^, albeit selectively for fearful faces while there was no effect for happy faces. Negative memory bias is consistently reported in depressed and dysphoric participants with better memory performance for sad faces than for happy faces^38–40^. However, the present results cannot be explained by subtle depressive symptoms as indexed by statistical analysis of the ADS-K questionnaire (see supplementary material ADS-K analysis).

### Facial feedback manipulation influences memory

Importantly, our results indicate that FFM systematically influences memory performance for facial emotional stimuli, at least in men. In contrast to the control manipulation, the smiling manipulation improved the memory performance selectively for happy faces and induced a positive bias independent of emotional quality.

Numerous previous studies have demonstrated that facial mimicry and the resulting facial feedback are both important for the processing of emotional stimuli in general^41^ and for the perception of facial emotional expressions in particular^13–15,22,42–44^. It is thought that facial mimicry supports embodied simulation processes: the perception of an emotional expression results in an internal simulation of a relating affective state via the activation of corresponding motor, somatosensory, affective and reward systems. This in turn helps to understand and interpret the expression^45^.

To date, research assessing the influences of facial feedback on emotional memory is scarce. We can hence only speculate about the processes underlying the link between facial muscle activation and memory:

Possibly, the observed effect of FFM is related to general mood modulation processes. The smiling manipulation may thus have activated the corresponding affective system in the participants and consequently resulted in a positive mood, which in turn helps to store congruent information in memory. Previous studies consistently demonstrated that FFM can systematically induce and modulate mood^46–48^. Further, some evidence suggests that mood itself can influence memory performance^49,50^. A mood-congruent memory effect is additionally supported by results demonstrating the tendency to better recall information that is congruent to the current mood in depressed and anxious participants^38,51,52^. In the present study, we assessed the influence of FFM on the participants’ affect by asking them to complete the IPANAT^32^ before starting with the paradigm, midway after a smiling manipulation block, and at the end of the experiment (depending on the version of the paradigm either after a hand or after a teeth condition). Indeed, smiling significantly decreased participants’ negative affect while the positive affect remained unchanged (see supplementary material). However, as the IPANAT does not measure explicit mood but rather affective trait and state, it provides only indirect evidence for how the FFM might have influenced the mood of the participants. While there is some evidence suggesting that the FFM modifies mood, the three measurements taken in the present study do not permit unambiguous proof for the effects of facial feedback on emotional memory performance. Therefore, in future research, it would be interesting to systematically measure the influence of facial feedback on mood changes.

The FFM effect might also be attributable to changes in processing style. There is compelling evidence that happy mood triggers a global and automatic processing style while sad mood triggers more local and analytic processing^53–55^. Following this, the smiling manipulation might have caused a positive mood and consequently triggered a more global and automatic processing style in the participants. However, in the present task, which asked for memorizing facial emotional expressions at different intensity levels, a local, more analytical processing style might have been more favorable than a global automatic processing style, to allow for the processing of more subtle differences between the intensity levels. Thus, while the present FFM might have evoked mood changes, these mood changes may not fully explain the reduced bias observed during the smiling FFM.

Alternatively, the influence of FFM might also be explained on a more neural level. As mentioned above, the reactivation account of memory assumes that remembering a piece of information activates the same brain regions that were engaged during the encoding phase. One might speculate that the FFM in the present study primed the related brain regions which were activated during a smiling expression and—most importantly—which were also activated during the storage and the retrieval of related information like the memory of a smiling face. In the past, imaging studies provided insights into the brain regions that are involved in WM processes of emotional faces^56–59^. These studies found activation within frontal areas, especially within the dorsolateral prefrontal and orbitofrontal cortex (dlPFC, OFC), as well as within the superior temporal sulcus (STS) and the amygdala. The dlPFC plays a fundamental role within the WM network^60–63^. Both the OFC and the amygdala contain face-selective neurons^64,65^ and their connective activity is thought to be responsible for differentiating positive and neutral facial expressions from negative ones^66^. Further, amygdala activation is allegedly related to enhanced memory for emotional stimuli^67–71^. Finally, the STS is a well-known structure in processing changeable features of faces such as emotional expressions^72,73^. Previous research revealed that the activation of the amygdala, hippocampus (especially right) and STS are related to facial mimicry processes during the perception of emotionally expressive faces^43,44,74–77^. It is thought that the activation of the right hippocampus displays the recruitment of memory contents for an improved understanding of the displayed facial expression^76^ while STS activation presents not only the sensory representation of the visual information but also an emotional communication process^77^. Thus, the FFM possibly primed the activation of those brain regions engaged during emotional memory processing and consequently facilitated the storage and retrieval of related information about facial expressions. With respect to future research, it would be interesting to shed further light on the activity of related brain regions during memorizing facial emotional expressions and the contribution of facial feedback to those processes.

### Gender difference

Our data show that men were more susceptible to the FFM. They remembered emotional expressions to be less negative/more positive in the teeth compared to the control manipulation condition, thereby reducing the negative bias. An analogous gender effect has recently been shown by Wood and colleagues in 2019^78^. There, the recognition of facial expressions and hand gestures was impaired after facial mobility restriction in men but not in women. Further evidence that male participants are more susceptible to FFMs comes from a study looking at pacifier use in childhood^8^. This study revealed a negative correlation between the duration of pacifier use and the amount of facial mimicry in boys but not in girls and that this effect seems to further impact social skills of men in later life. Especially skills that depend on the recognition of others’ emotion were affected.

Meanwhile, women generally outperform men during emotion perception tasks, with a more pronounced advantage for negative emotions (for review see Thompson and Voyer^79^). This advantage can be of biological as well as cultural origin—women as caregivers are more in demand of recognizing negative emotions^79^ and women as “emotion experts” profit of particular emotional stimulation in childhood^80,81^. Finally, there exists some evidence suggesting that women are more responsive towards emotional facial expressions in their own facial reactions^1^ and generally show more emotional expressions than men and tend to smile more^82^. Consequently, it might be that in the present study female participants reached a ceiling effect regarding the potential influence of FFM while male participants did not exploit their facial mimicry and expressivity to its full potential and could thus profit from the manipulation.

Since previous studies did not reveal gender differences in the influence of a smiling manipulation on emotion perception^13,15^ it is unlikely that our results rely on influences of perceptual processes only. Additionally, a sole influence of feedback manipulation on perceptual processes would have affected the target as well as the test image and both should have been perceived as more positive. As a consequence, such general perception biases should have cancelled each other out. Therefore, while it is possible that the undertaken FFM influenced the perception of the emotional faces, this could not fully explain the present results. Notwithstanding the female advantage in emotion recognition abilities, a gender difference in memorizing abilities for facial emotional expressions remains incompletely understood and should be topic of future studies.

### Limitations and further directions

There are limitations in this study that should be addressed in future research. First, because of the implementation of the FFM (holding a pen with the non-dominant hand vs. holding it with the teeth) the manipulation was maintained during the whole duration of the testing session, alternating between control and smiling blocks. For this reason, data do not allow for a detailed separation of the influence on the different stages of memory processing. We therefore cannot confidently assert whether the performed FFM influenced the storage, encoding, maintenance or the retrieval of emotional faces. Based on the present data, future studies should apply a more specific FFM, either during target or test image presentation. Second, in contrast to previous studies^13,24,42^ and to the seminal FFM study^16^, we did not assess the influence of a pen-between-lips condition in the present study. Since previous studies have shown only minor to no effects of this manipulation method^13,24^ and to ensure sufficient trial replications per condition and to prevent unnecessary exhaustion we decided to exclude this manipulation condition. Using the pen between the teeth condition, we found that posing a smile affected the memory processes of emotional faces. We are hence convinced that the omission of the pen between the lips condition does not take away from our most important finding, that facial feedback from the smiling muscle (zygomaticus major) results in more positive memory to emotional facial expressions. It will have to be the aim of future research to specifically compare the effects between a frowning and a smiling manipulation condition.

A further limitation is related to the task specificity. In the present paradigm participants had to remember the facial expression and the intensity of this expression, allowing to investigate the influence of FFM on memory for emotional faces. We cannot exclude that the manipulation might have also influenced the visual memory for more static aspects of the target such as identity or gender. Accordingly, future research of this topic should consider control tasks where participants have to remember the facial identity of a perceived face. Finally, as in the most of previous studies using the FFM technique, the relatively small number of participants might have resulted in a rather low statistical power.

## Conclusion

The present study examined the influence of FFM on memory for emotional faces. For this purpose we applied classical FFM where holding a pen with the teeth induced a smiling expression and thus increasing positive facial feedback while holding it with the non-dominant hand served as control condition. Facial feedback of the participants was manipulated while they performed a visual WM paradigm where they had to remember the intensity of either a happy or sad facial emotional expression. Data show that the smiling manipulation condition improved memory performance selectively for happy faces, especially when highly ambiguous facial expressions had to be remembered. Furthermore, we found that FFM induced a general positive bias (and reduced the negative memory bias) for the remembered emotional facial information. Finally, data demonstrated that men were more affected by the FFM than women.

The influences of the smiling manipulation might be attributed to the priming of activation of a specific brain network engaged during memory processes for emotional faces. Consequently, this priming might facilitate the storage and retrieval of congruent information. Our data demonstrate that facial feedback not only influences perception but also systematically alters memory of the facial emotional expressions. This study constitutes a first step towards our understanding of the influence of facial feedback on memory of emotional facial expressions.

## Supplementary information

Supplementary Information.

## Data Availability

The conditions of our ethics approval do not permit public archiving of the data. Readers seeking access to the data should contact the corresponding author. Requestors must complete a formal data sharing agreement to obtain the data. The experimental code used in this study is available at https://osf.io/kb92n/. No part of the study procedures or analyses was pre-registered prior to the research being conducted.
