# Supplementary material for: Effects of posed smiling on memory for happy and sad facial expressions
Source: Sci Rep. 2021 May 18;11:10477. doi: 10.1038/s41598-021-89828-7 (PMC8131584; doi:10.1038/s41598-021-89828-7)
Supplement: Supplementary file 1 — Supplementary Information. [file 41598_2021_89828_MOESM1_ESM.docx]

**Supplementary Material**

***Emotional bias formulas***

- Happy target face correctly remembered as happy face

$$\boldsymbol{Happy}_{\boldsymbol{correct}}=\% test image-\% sample image$$

Example Sample image: 40% happy Test image: 30% happy

Emotional bias: 40% - 30% = 10%

- Happy target face incorrectly remembered as sad face

$$\boldsymbol{Happy}_{\boldsymbol{wrong}}= -\left( \% sample image+\% test image \right)$$

Example Sample image: 40% happy Test image: 30% sad

Emotional bias: -(40% + 30%) = -70%

- Sad face correctly remembered as sad face

$$\boldsymbol{Sad}_{\boldsymbol{correct}}=\% sample image-\% test image$$

Example Sample image: 30% sad Test image: 40% sad

Emotional bias: 30% - 40% = -10%

- Sad face incorrectly remembered as sad face

$$\boldsymbol{Sad}_{\boldsymbol{wrong}}=\% sample image+\% test image$$

Example Sample image: 30% sad Test image: 20% happy

Emotional bias: 30% + 20% = 50%

***Influence of depressive symptoms***

The ADS-K (Allgemeine Depressionsskala, short-version) is a self-report questionnaire measuring impairments caused by depressive symptom during the previous weeks. The present data show that participants exhibited an overall negative bias for happy faces compared to sad faces. To exclude any influence of depressive symptomatic on this negative bias we conducted an additional statistical analysis. For this purpose, a RM-ANOVA with *facial feedback manipulation* and *emotion* as within- and gender as between-participant factors and *ADS-K* as covariate was conducted. Analogously to the main results, this ANOVA revealed a significant main effect of *facial feedback manipulation* (F_1,34_ = 4.148, P = 0.05, $\eta_{p}^{2}$ = 0.109). Further the interactions of *emotion* x *ADS-K* as well as *facial feedback manipulation* x *emotion* x *ADS-K* did not reach significance (all Ps > 0.8) and there was no main effect of *ADS-K* (F1,34 = 0.131, P = 0.720, $\eta_{p}^{2}$ = 0.004). Consequently, it can be ruled out that the apparent negative bias is caused by the influence of any depressive symptoms of the participants.

***Influence of emotional state***

The IPANAT (Implicit Positive and Negative Affect Test) is a self-report questionnaire of measuring the negative and positive affect (Quirin et al., 2009). To control for the impact of our facial feedback manipulation on the emotional state of the participants we performed an additional statistical analysis. Two separate paired t-tests were calculated to directly compare both the positive and negative affect in the teeth condition with that before participants ran the experiment as control condition. This analysis revealed a significant difference for the negative affect between these two measurement time points, where the negative affect was significantly decreased after the teeth (M = 1.68, SD = 0.42) compared to the control condition (M = 1.82, SD = 0.51, t(36) = 2.326, P = 0.026, d = 0.3). However, the positive affect of the participants did not differ between the two time points (M_control_ = 2.15, SD_control_ = 0.40, M_teeth_ = 2.24, SD_teeth_ = 0.41, t(36) = -1.501,P = 0.142, d = -0.222). Thus, during the smiling condition the negative affect was significantly decreased compared to the control condition, indicating that the emotional affect of the participants was modulated by our facial muscle manipulation.

***Emotional bias excluded trails***

During the analysis of the emotional bias, values exceeding ±2 standard deviations from the mean were excluded. Number of excluded trials did not differ between facial feedback manipulation, emotion and gender (Ps>.05).

**Table S1.** Number of excluded trials

|  | Hand | |  | teeth | |
| --- | --- | --- | --- | --- | --- |
|  | happy | sad |  | happy | sad |
| men | 2.83 (1.07) | 3.39 (1.16) |  | 2.72 (1.10) | 3.33 (1.29) |
| women | 2.84 (0.81) | 3.11 (0.97) |  | 2.58 (1.14) | 2.79 (1.24) |

Number of excluded trials (M(SD) separately for men and women over the both facial feedback manipulation conditions for happy and sad faces.
